# Prognostic significance of platelet-to-lymphocyte ratio in urothelial carcinoma patients: a meta-analysis

**DOI:** 10.1186/s12935-019-1032-6

**Published:** 2019-11-27

**Authors:** Yuhai Bao, Yin Wang, Xiaodong Li, Mingjun Pan, Hongze Zhang, Zegen Cheng, Xueyi Wang

**Affiliations:** Department of Urology, General Hospital of Benxi Iron & Steel Industry Group of Liaoning Health Industry Group, Benxi, 117000 Liaoning China

**Keywords:** Upper tract urothelial carcinoma, Platelet-to-lymphocyte ratio, Meta-analysis, Survival, Tumor stage

## Abstract

**Background:**

The prognostic value of pre-treatment platelet-to-lymphocyte ratio (PLR) in patients with urothelial carcinoma (UC) remains controversial. Therefore, this meta-analysis aimed to identify the prognostic impact of PLR on UC.

**Methods:**

The PubMed, Embase, Web of Science, and Cochrane Library databases were systematically searched. Hazard ratios (HRs) with 95% confidence intervals (CIs) were used to summarize the correlations between PLR and overall survival (OS), progression-free survival (PFS), disease-free survival (DFS), and cancer-specific survival (CSS). Odds ratios (ORs) with 95% CIs were used to measure the association between PLR and tumor clinicopathological factors.

**Results:**

The meta-analysis included 15 studies published from 2015 to 2019 with a total of 5354 patients. Overall, a high PLR was correlated to poorer PFS (HR = 1.81, 95% CI 1.28–2.56, p = 0.001) and DFS (HR = 1.09, 95% CI 1.31–2.16, p < 0.001) but not poor OS (HR = 1.23, 95% CI 0.95–1.59, p = 0.124) or CSS (HR = 1.000, 95% CI 0.998–1.002, p = 0.919) in UC. In addition, an elevated PLR was correlated with patient age > 65 years (OR = 1.72, 95% CI 1.25–2.38, p = 0.001) and hypertension (OR = 1.48, 95% CI 1.01–2.18, p = 0.046). However, no significant association was observed between PLR and sex (OR = 0.79, 95% CI 0.56–1.14, p = 0.206) or diabetes (OR = 1.29, 95% CI 0.77–2.15, p = 0.333).

**Conclusions:**

Our results demonstrated a significant correlation between elevated PLR and poor prognosis in UC. The prognostic role of PLR may help guide the management and prognostication of UC patients.

## Background

Urothelial carcinomas (UCs) are the fourth most prevalent tumors [1]. Upper tract urothelial carcinomas (UTUC) are tumors derived from the urothelium along the urinary tract [2]. UTUCs are rare, accounting for only 5–10% of all UCs [3, 4], while bladder cancer (BC) accounts for 90% of all UCs. Sixty percent of UTUCs are diagnosed at the invasive stage, and peak incidence is observed in patients aged 70–90 years [5]. Regardless of the tumor location in the upper urinary tract, radical nephroureterectomy (RNU) with bladder cuff resection is considered the standard treatment for most UTUC patients [5]. Although an adequate surgical treatment, the 5-year cancer-specific mortality remains high, ranging from 20% to 30% [6]. Seventy-five percent of BC patients are diagnosed with non-muscle-invasive bladder cancer (NMIBC), which has a high risk of recurrence. Various prognostic factors such as p53 protein, nuclear factor-kB, and osteopontin have been investigated in UC, but the prognostic efficiency remains unsatisfactory [2]. Therefore, it is important to identify reliable and effective prognostic biomarkers to aid UC prognostication and treatment.

Recent studies have shown that inflammation and immune responses play a role in cancer development [7–9]. The systemic inflammatory response (SIR) can substantially influence UC progression [10–12]. A series of hematological parameters, reflecting the immune status of cancer patients, have been widely explored as prognostic markers in recent years [13–15]. Neutrophil-to-lymphocyte ratio (NLR), lymphocyte-to-monocyte ratio (LMR), and platelet-to-lymphocyte ratio (PLR) are non-invasive and cost-effective prognostic indicators for solid tumors [16–19]. Recent retrospective studies have reported inconsistent findings regarding the prognostic impact of PLR in UC [11, 12, 20–32]. For example, some studies reported a positive association between a high PLR and poor survival in UC [24, 29], whereas others did not [20] or even showed the opposite trend [12, 30]. Therefore, the present meta-analysis aimed to estimate the prognostic role of PLR for different survival outcomes in UC. Furthermore, the associations between PLR and various clinicopathological factors were also analyzed.

## Materials and methods

### Search strategy

The PubMed, Embase, Web of Science, and Cochrane Library electronic databases were systematically searched to identify relevant studies. The following terms were used in the literature search: “platelet lymphocyte ratio”, “PLR”, “platelet to lymphocyte ratio”, “urothelial carcinoma”, “urothelial cancer”, “bladder cancer”, “bladder tumor”, “upper urinary tract cancer”, “upper tract urothelial carcinoma”, and “UTUC”. The last search was updated on September 16, 2019. The reference lists of relevant articles were also examined for additional potential inclusions. This meta-analysis was performed in accordance with the Preferred Reporting Items for Systematic Reviews and Meta-analyses (PRISMA) statement [33]. Ethical approval and informed consent were waived because all studies included in this meta-analysis were previously published.

### Inclusion criteria and exclusion criteria

The inclusion criteria were (1) pathologically or histologically confirmed diagnosis of UC; (2) studies evaluating the correlation between PLR and overall survival (OS), progression-free survival (PFS), disease-free survival (DFS), and/or cancer-specific survival (CSS); (3) defined PLR cut-off value; (4) preoperative blood cell counts; (5) hazard ratio (HR) and 95% confidence interval (CI) provided or able to be calculated from the available information; and (6) studies published as full-text in English. The exclusion criteria were (1) case reports, reviews, meeting abstracts, or letters; (2) studies with overlapping or duplicate data; and (3) studies without sufficient or usable data.

### Data extraction

Two investigators (Y.B and Y.W) independently reviewed all candidate studies, and any disagreements were resolved by discussion with a third investigator (X.L). The following information was extracted from each eligible study: first author, year of publication, country, study period, number of patients, sex, age, treatment, cut-off value, ethnicity, survival analysis, and HRs of PLR for OS, PFS, DFS, and CSS with their 95% CIs.

### Quality assessment

Study quality was evaluated using the Newcastle–Ottawa scale (NOS) [34]. The NOS consists of three parts: selection, outcome, and comparability. The scores range from 0 to 9, and studies with NOS scores ≥ 6 are considered to be high-quality studies.

### Statistical analysis

This meta-analysis was conducted using Stata 12.0 (Stata Corp, College Station, TX, USA). Heterogeneity among studies was estimated using Cochran’s Q test [35] and Higgins’ I-squared statistics [36]. A random‐effects model was used for studies with significant heterogeneity (*I*^2^ > 50% or Ph < 0.10). Otherwise, a fixed-effects model was used. A pooled HR > 1 with 95% CI not overlapping 1 (p < 0.05) indicated worse OS, PFS, DFS, and CSS for a high PLR in UC. Subgroup analyses were performed to investigate the factors influencing the prognostic function of PLR. The correlation between PLR and clinicopathological factors were measured by pooled odds ratios (ORs) and 95% CIs. Publication bias was determined using Begg’s funnel plots and Egger’s linear regression tests. p-values < 0.05 were considered statistically significant.

## Results

### Selection and characteristics of the included studies

**Fig. 1:**
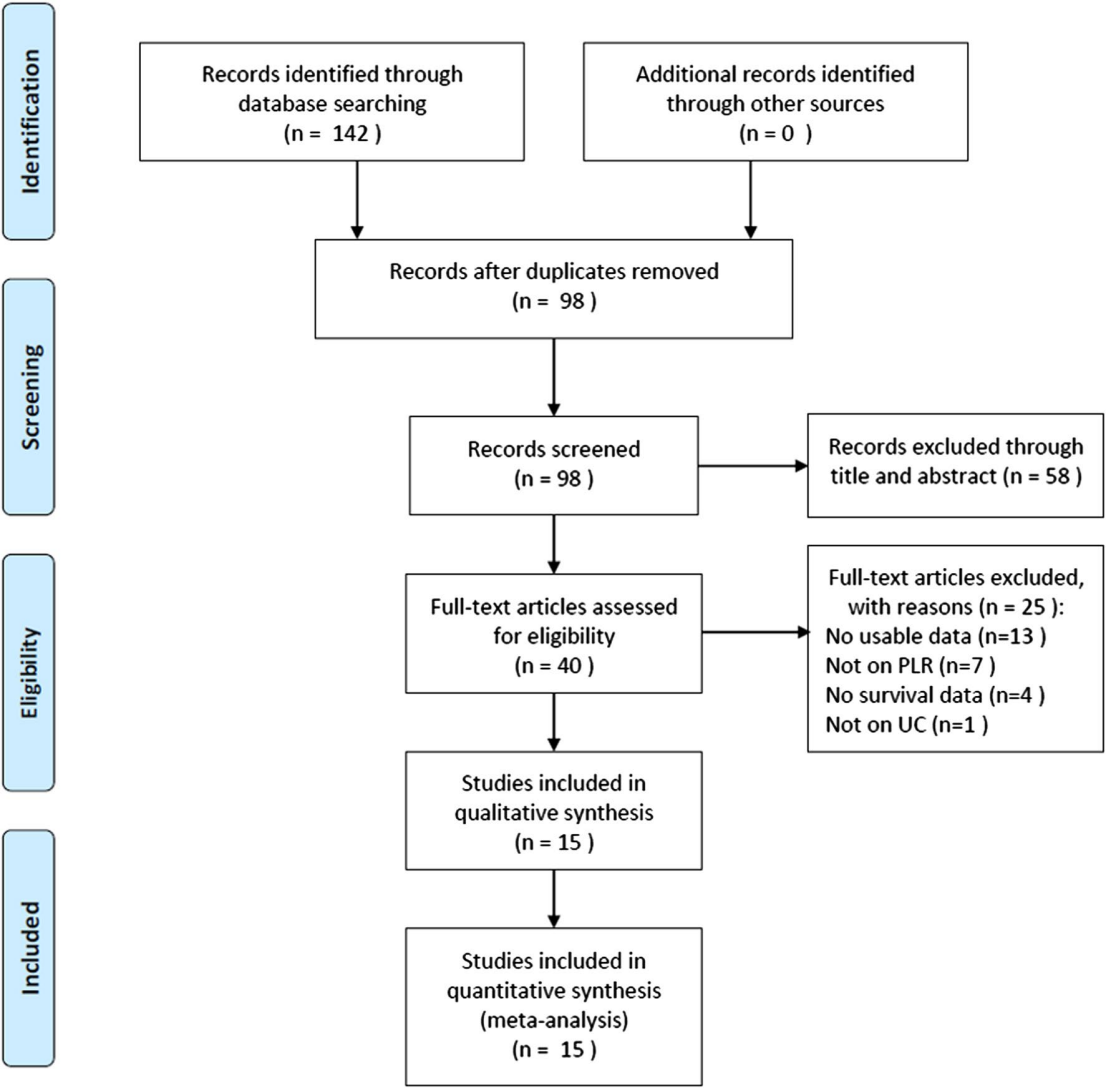
The flow diagram indicated the process of study selection

The process of literature selection is shown in Fig. 1. The initial literature search identified 142 studies and excluded 44 duplicate records. The remaining 98 studies were screened by title and/or abstract, and 58 studies were excluded. Subsequently, 40 full-text articles were evaluated, and 25 studies were removed for the following reasons: insufficient information (n = 13), not involving PLR (n = 7), no survival data (n = 4), and not involving UC (n = 1). Finally, 15 studies [11, 12, 20–32] with a total of 5354 patients were included in the meta-analysis. The major characteristics of the 15 eligible studies are presented in Table 1. These studies were published from 2015 to 2019 and were conducted in six countries including China (n = 5) [21, 22, 25, 27, 32], Korea (n = 4) [20, 26, 29, 31], Japan (n = 3) [11, 12, 30], Austria (n = 1) [24], Poland (n = 1) [28], and Turkey (n = 1) [23]. The sample sizes ranged from 113 to 1551, with a median value of 186. The cut-off PLR values varied from 111 to 241. Eight studies [12, 20, 22–25, 29, 30] investigated the prognostic value of PLR in UTUC while seven studies focused on BC [11, 21, 26–28, 31, 32]. Regarding the prognostic role of PLR in UC, nine studies reported OS [11, 12, 21, 24–26, 28, 31, 32], seven studies reported PFS [22–25, 27, 29, 30], six studies reported DFS [20, 22, 23, 27, 29, 30], and five studies provided data on CSS [11, 20, 26, 28, 31]. All studies had NOS scores ≥ 6.

**Table 1:** The basic information and data of all included studies in the meta-analysis

Author	Year	Country	No. of patients	Sex (M/F)	Tumor type	Age (years) Median (range)	Cut-off value	Survival analysis	Treatment	NOS score
Kim	2015	Korea	277	218/59	UTUC	63.7 (29.5–90)	150	CSS, DFS	NUx	7
Zhang	2015	China	124	100/24	BC	65 (30–78)	140	OS	RC	8
Song	2016	China	140	86/54	UTUC	67 (39–81)	128	DFS, PFS	RNU	6
Altan	2017	Turkey	113	86/27	UTUC	63.7	150	DFS, PFS	RNU	7
Dalpiaz	2017	Austria	180	109/71	UTUC	70	150	OS, PFS	RNU	8
Huang	2017	China	481	311/170	UTUC	65 (30–89)	241	OS, PFS	RNU	7
Kang	2017	Korea	1551	1302/249	BC	65 (57–72)	124	OS, CSS	TURB	7
Mao	2017	China	207	169/38	BC	66 (59–80)	123	DFS, PFS	TURB	8
Miyake	2017	Japan	117	95/22	BC	72	150	OS, CSS	RC	8
Rajwa	2018	Poland	144	NA	BC	NA	161	OS, CSS	RC	6
Son	2018	Korea	1137	825/312	UTUC	69	142	DFS, PFS	RNU	6
Itami	2019	Japan	125	96/29	UTUC	72 (38–90)	150	OS, DFS	NUx	7
Kuroda	2019	Japan	187	138/49	UTUC	71 (38–90)	165	DFS, PFS	RNU	7
Yuk	2019	Korea	385	327/58	BC	72.6	171	OS, CSS	TURB	7
Zhu	2019	China	186	157/29	BC	65	111	OS	RC	7

*OS* overall survival, *DFS* disease-free survival, *PFS* progression-free survival, *CSS* cancer-specific survival, *NUx* nephroureterectomy, *RC* radical cystectomy, *RNU* radical nephroureterectomy, *TURB* transurethral resection of bladder tumor, *NA* not available, *NOS* Newcastle–Ottawa Scale, *UTUC* upper tract urothelial carcinoma, *BC* bladder cancer

### Impact of PLR on OS, PFS, DFS, and CSS

**Fig. 2:**
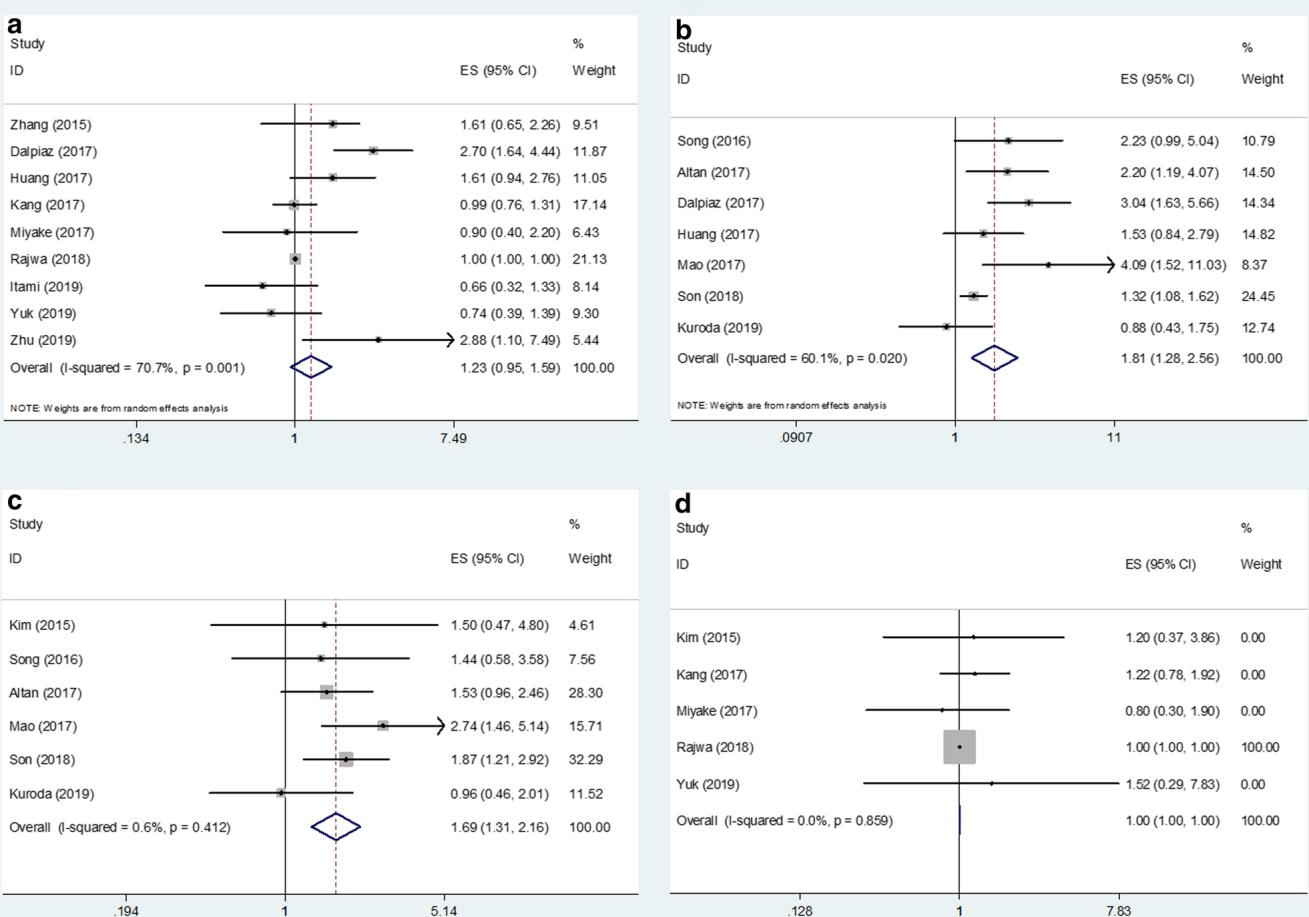
Forest plots of studies evaluating the effect of elevated PLR on the HR and 95% CI of **a** OS **b** PFS **c** DFS, and **d** CSS in UC patients

Nine studies [11, 12, 21, 24–26, 28, 31, 32] provided data on the relationship between PLR and OS. The pooled HR and 95% CI were: 1.23 and 0.95–1.59, respectively (p = 0.124) (Table 2, Fig. 2), indicating that PLR was not a significant marker for OS. Subgroup analysis showed that PLR was associated with worse OS in patients receiving radical nephroureterectomy (RNU) (HR = 2.13, 95% CI 1.48–3.07, p < 0.001; Table 2), although this subgroup included only two studies. Seven studies [22–25, 27, 29, 30] reported the impact of PLR on PFS. The pooled HR and corresponding 95% CI were 1.81 and 1.28–2.56, respectively (p = 0.001) (Table 2, Fig. 2). Subgroup analysis showed that PLR remained a significant indicator for PFS irrespective of sample size and tumor type. Six studies [20, 22, 23, 27, 29, 30] evaluated DFS. The pooled HR was 1.09 (95% CI 1.31–2.16, p < 0.001) (Table 2, Fig. 2). The subgroup analysis demonstrated that PLR remained correlated with poor DFS in patients with UTUC and BC as well as patients receiving RNU and transurethral resection of bladder tumor (TURB) (Table 2). Five studies provided CSS data [11, 20, 26, 28, 31] with pooled HR and 95% CI of 1.000 and 0.998–1.002, respectively (p = 0.919). The subgroup analysis indicated that PLR was not associated with CSS regardless of sample size, tumor type, or treatment method.

**Table 2:** Subgroup analysis of the relationship between PLR and OS, PFS, DFS, and CSS in UC

Factors	No. of studies	Effect model	HR (95%CI)	p	Heterogeneity	
					*I*^2^(%)	Ph
OS						
All	9	Random	1.23 (0.95–1.59)	0.124	70.7	0.001
Sample size						
≤200	6	Random	1.37 (0.87–2.15)	0.174	78.7	<0.001
>200	3					
Tumor type						
UTUC	3	Random	1.47 (0.70–3.12)	0.309	80.2	0.006
BC	6	Fixed	1.002 (1.000–1.004)	0.049	36.1	0.166
Treatment						
RC	4	Random	1.27 (0.83–1.95)	0.266	56.8	0.074
RNU	2	Fixed	2.13 (1.48–3.07)	<0.001	47.6	0.167
TURB	2	Fixed	0.95 (0.74–1.22)	0.666	0	0.409
NUx	1	–	0.66 (0.32–1.35)	0.253	–	–
PFS						
All	7	Random	1.81 (1.28–2.56)	0.001	60.1	0.020
Sample size						
≤200	4	Random	1.92 (1.14–3.26)	0.015	58	0.067
>200	3	Random	1.68 (1.03–2.75)	0.040	60.1	0.020
Tumor type						
UTUC	6	Random	1.67 (1.2–2.31)	0.002	54.8	0.050
BC	1	–	4.09 (1.52–11.03)	0.005	–	–
DFS						
All	6	Fixed	1.69 (1.31–2.16)	<0.001	0.6	0.412
Sample size						
≤200	3	Fixed	1.35 (0.94–1.95)	0.103	0	0.573
>200	3	Fixed	2.05 (1.45–2.90)	<0.001	0	0.535
Tumor type						
UTUC	5	Fixed	1.54 (1.17–2.02)	0.002	0	0.672
BC	1	–	2.74 (1.46–5.14)	0.002	–	–
Treatment						
RNU	4	Fixed	1.54 (1.17–2.04)	0.002	0	0.504
TURB	1	–	2.74 (1.46–5.14)	0.002	–	–
NUx	1	–	1.50 (0.47–4.80)	0.499	–	–
CSS						
All	5	Fixed	1.000 (0.998–1.002)	0.919	0	0.859
Sample size						
≤200	2	Fixed	1.000 (0.998–1.002)	0.923	0	0.635
>200	3	Fixed	1.23 (0.82–1.85)	0.311	0	0.968
Tumor type						
UTUC	1	–	1.20 (0.37–3.86)	0.757	–	–
BC	4	Fixed	1.000 (0.998–1.002)	0.919	0	0.748
Treatment						
RC	2	Fixed	1.000 (0.998–1.002)	0.923	0	0.635
TURB	2	Fixed	1.24 (0.80–1.91)	0.334	0	0.801
NUx	1	–	1.20 (0.37–3.86)	0.757	–	–

### Correlations between PLR and clinicopathological factors in UC

**Fig. 3:**
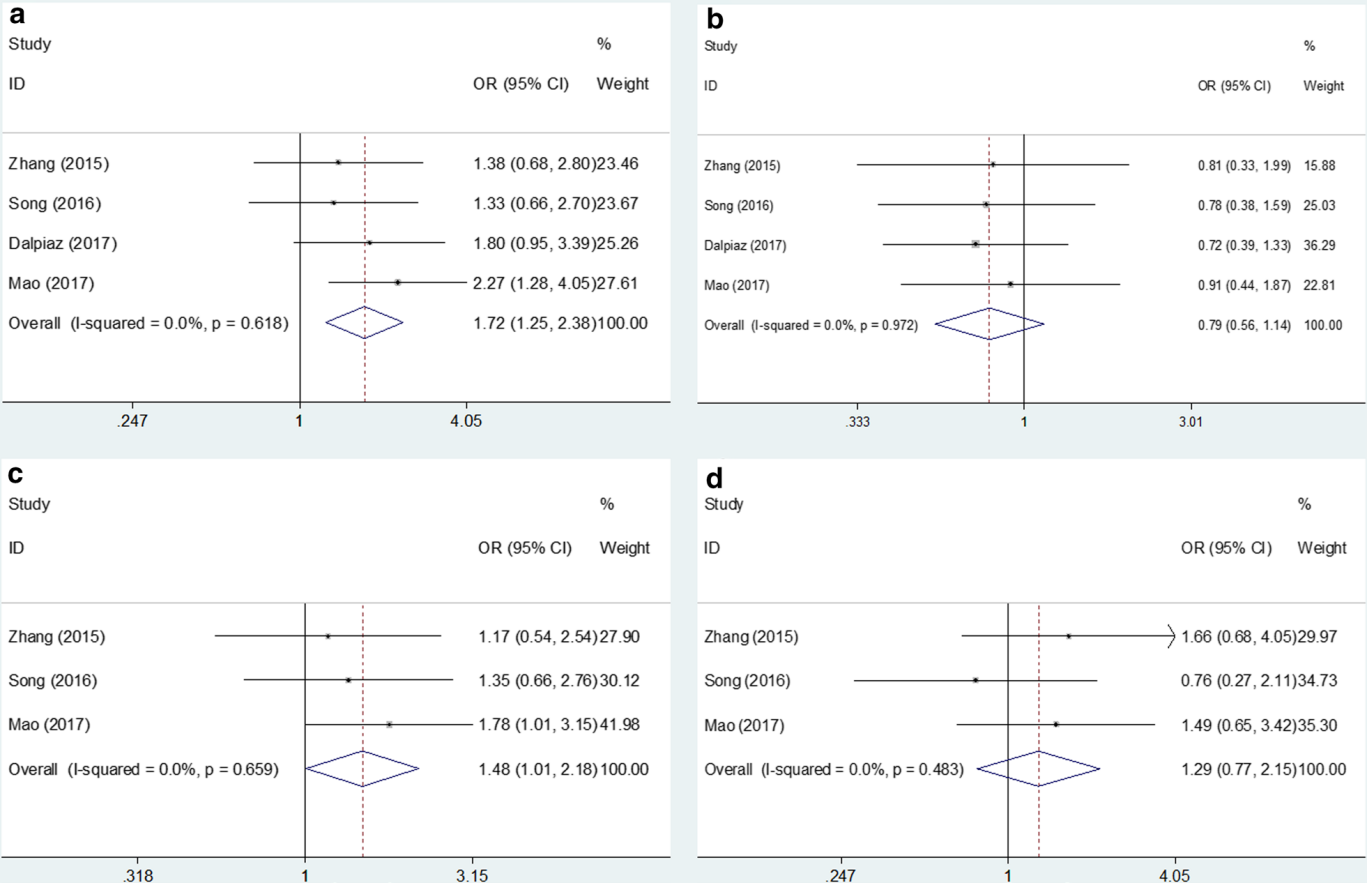
Forest plots of the association of PLR with clinicopathological parameters: **a** age (≥ 65 years vs < 65 years) **b** sex (male vs female) **c** hypertension (yes vs no), and **d** diabetes (yes vs no)

Four studies [21, 22, 24, 27] provided relevant data on the associations between PLR and clinicopathological characteristics. The associations between PLR and clinical factors were calculated using pooled ORs. As shown in Fig. 3, the pooled ORs and 95% CIs indicated that a high PLR was correlated to patient age > 65 years (OR = 1.72, 95% CI 1.25–2.38, p = 0.001; Fig. 3) and hypertension (OR = 1.48, 95% CI 1.01–2.18, p = 0.046; Fig. 3). However, no significant association was found between PLR and sex (OR = 0.79, 95% CI 0.56–1.14, p = 0.206) or diabetes (OR = 1.29, 95% CI 0.77–2.15, p = 0.333) (Fig. 3).

### Publication bias

Publication bias was examined by both Begg’ s and Egger’s tests. We found no significant publication bias in OS (p = 0.917 for Begg’s test; p = 0.209 for Egger’s test), PFS (p = 0.230 for Begg’s test; p = 0.131 for Egger’s test), DFS (p = 0.851 for Begg’s test; p = 0.599 for Egger’s test), or CSS (p = 0.462 for Begg’s test; p = 0.368 for Egger’s test).

## Discussion

The present study comprehensively searched relevant databases and collected data from 15 cohort studies containing 5354 patients. The pooled results suggested that an elevated PLR predicted an inferior PFS and unfavorable DFS in UC patients. The subgroup analysis showed that the prognostic value of PLR for DFS and PFS was not influenced by tumor type. Moreover, the pooled data also indicated that a high PLR was associated with patient age > 65 years and hypertension. To our knowledge, ours is the first meta-analysis to investigate the prognostic ability of PLR in patients with UC. As PLR is non-invasive and easily accessible, it has the potential to guide clinical decision-making.

Recent studies using meta-analytic methods also focused on the association between PLR and prognosis for various types of cancer [17]. Lin et al. showed that a high pretreatment PLR predicted worse OS (HR = 1.73, 95% CI 1.46–2.04, p < 0.001) and DFS (HR = 1.30, 95% CI 1.06–1.60, p = 0.01) in hepatocellular carcinoma patients with different Barcelona Clinic Liver Cancer (BCLC) stages [37]. Similarly, Wang et al. also reported an association between an elevated PLR and poor OS (HR = 1.85, 95% CI 1.51–2.25, p < 0.001) as well as DFS (HR = 1.4, 95% CI 1.1–1.79, p = 0.007) in prostate cancer patients [38]. Another work showed the prognostic value of PLR for worse OS (HR = 1.38, 95% CI 1.19–1.62, p < 0.001) and poor RFS or PFS (HR = 1.55, 95% CI 1.27–1.88, p < 0.001) in patients with cholangiocarcinoma [39]. The findings of previous studies were in line with those of the current study. The present study computed the prognostic value and clinical significance of PLR using pooled HRs and ORs. For this reason, the results might not be applicable to individual patients because platelet and lymphocyte counts are influenced by multiple factors such as infection, inflammation, drug use, age, and baseline physical condition. Therefore, when applying PLR for prognostication of individual patients with UC, other clinicopathological factors should also be considered. Furthermore, cell counts performed at different sites could vary, which makes it difficult to normalize PLR.

The exact mechanisms by which PLR has prognostic value in UC patients remain unclear. Cancer cells can induce platelet activation by secreting platelet agonists [40]. Platelets also facilitate the proliferation of ovarian cancer cells in a transforming growth factor-β1 (TGF-β1)-dependent manner [41]. Moreover, platelets can directly contact tumor cells and secret a series of cytokines including platelet-derived growth factor (PDGF), TGF-β, and prostaglandin (PG) E2, which can enhance the epithelial-mesenchymal transition (EMT) of tumor cells [42, 43]. In contrast, lymphocytes play important roles in anti-tumor immune responses. Intraepithelial CD3 + and CD8 + tumor-infiltrating T lymphocytes (TILs) were strongly associated with improved PFS and DFS in ovarian cancer patients [44]. Lymphocytes and interferon (IFN) gamma can collaborate to select to tumor cells to reduce immune surveillance [45]. CD8 + TILs have been associated with good prognosis in various cancers [46]. Therefore, evaluation of PLR is useful and convenient to predict clinical outcomes in patients with UC.

The present study had several limitations. First, the included studies were all retrospective, which may have caused a selection bias in the meta-analysis. Second, only four studies provided data on the association between PLR and clinical features. The sample size was too small. Third, we extracted pooled HRs and 95 CIs from eligible studies but not individual patient information. Fourth, it is hard to normalize PLR because blood counts may vary at different sites, which may cause variability in the index values. Therefore, additional large-scale prospective studies are warranted to confirm our findings.

## Conclusions

The results of this meta-analysis showed that PLR predicted worse DFS and PFS in UC. PLR was also correlated with older age and hypertension in patients with UC. The prognostic role of PLR may help to guide the administration of treatment and prognostication of UC patients.

## Data Availability

The data that support the findings of this study are available from the corresponding author upon reasonable request.

## Abbreviations

PLR: platelet-to-lymphocyte ratio
UC: urothelial carcinoma
UTUC: upper tract urothelial carcinoma
BC: bladder cancer
HR: hazard ratio
CI: confidence interval
OS: overall survival
DFS: disease-free survival
PFS: progression-free survival
UC: urothelial carcinoma
RNU: radical nephroureterectomy
SIR: systemic inflammatory response
NLR: neutrophil-to-lymphocyte ratio
LMR: lymphocyte-to-monocyte ratio
PRISMA: Preferred Reporting Items for Systematic Reviews and Meta-analyses
NOS: Newcastle–Ottawa scale
